# Concurrent validity of the Ages and Stages Questionnaire Inventory and the Bayley Scales of Infant and Toddler Development in rural Bangladesh

**DOI:** 10.1186/s12887-022-03800-6

**Published:** 2023-03-01

**Authors:** Helen O. Pitchik, Fahmida Tofail, Fahmida Akter, Abul K. M. Shoab, Jesmin Sultana, Tarique M. N. Huda, Mahbubur Rahman, Peter J. Winch, Stephen P. Luby, Lia C. H. Fernald

**Affiliations:** 1 Division of Epidemiology, School of Public Health, University of California, 2121 Berkeley Way West, Berkeley, CA 94720 USA; 2 Nutrition and Clinical Services Division, Icddr,b, Dhaka, Bangladesh; 3 Infectious Diseases Division, Icddr,b, Dhaka, Bangladesh; 4 Department of International Health, Bloomberg School of Public Health, Johns Hopkins University, Baltimore, MD USA; 5 Division of Infectious Diseases and Geographic Medicine, Stanford University, Stanford, CA USA; 6 Division of Community Health Sciences, School of Public Health, University of California, Berkeley, CA USA

**Keywords:** Early child development, Early child assessment, Ages and Stages Questionnaire Inventory, Bayley Scales of Infant and Toddler Development

## Abstract

**Background:**

Reliable and valid measurement of early child development are necessary for the design of effective interventions, programs, and policies to improve early child outcomes. One widely used measure in low- and middle-income countries (LMICs) is the Bayley Scales of Infant and Toddler Development III (Bayley-III). Alternatively, the Bangladeshi-adapted Ages and Stages Questionnaire Inventory (ASQ:I) can be administered more quickly, inexpensively, and with less training than the Bayley-III. We aimed to assess the concurrent validity of the Bangladeshi-adapted ASQ:I with the Bayley-III in children 4–27 months old in rural Bangladesh.

**Methods:**

The sample was a sub-sample (*n* = 244) of endline participants from an evaluation of an early child development intervention (July–August 2018). We assessed concurrent validity between internally age-standardized domain-specific and total scores using Pearson correlations both overall and stratified by age and intervention status. We also assessed correlations between scores and variables theoretically related to child development including maternal education and stimulation in the home.

**Results:**

The overall correlation between ASQ:I and Bayley-III total scores was moderate (*r* = 0.42 95% CI: 0.30–0.53), with no systematic differences by intervention status. Overall, concurrent validity was highest for the gross motor domain (*r* = 0.51, 0.40–0.60), and lowest for the fine motor domain (*r* = 0.20, 0.04–0.33). Total ASQ:I and Bayley-III scores were positively correlated with child stimulation and maternal education.

**Conclusion:**

The Bangladeshi-adapted ASQ:I is a low-cost tool that can be feasibly administered in rural Bangladesh, is moderately correlated with the Bayley-III, and can be used to measure child development when human, time, or financial resources are constrained.

**Supplementary Information:**

The online version contains supplementary material available at 10.1186/s12887-022-03800-6.

## Introduction

Over 249 million children in low- and middle-income countries were at risk for poor development in 2010 [1]. Child development is a global priority, as demonstrated by the explicit inclusion of child development in the United Nations Sustainable Development Goal 4.2, “By 2030, ensure that all girls and boys have access to quality early childhood development, care and pre-primary education so that they are ready for primary education” [2]. The implementation and evaluation of early child development interventions is transitioning from small-scale interventions to large-scale delivery through routine health systems. Valid and feasible measurement of child development is important to understanding which interventions work to improve child development outcomes at-scale and track child development at the population level [3]. The tools used to evaluate child development outcomes following small scale interventions may no longer be feasible for the evaluation of large-scale interventions where financial, human, or time resources are limited.

Measurement tools to estimate children’s developmental status are either comprised of caregiver responses to questions about their child's attainment of developmental milestones (“caregiver report”) or direct assessments of child skills (“direct assessment”) [4]. Direct assessments include test items that are administered directly to the child by a trained assessor, and caregiver report assessments are administered as a questionnaire that is either filled out directly by the child’s primary caregiver or as an interview with the child’s primary caregiver. Direct assessments are thought to be less biased and more precise compared to caregiver report, especially when the assessments are used for the evaluation of intervention impacts [4]. If assessors are masked to intervention status, direct assessments avoid the potential bias from differential caregiver report depending on intervention status. Direct assessments may also more precisely determine children’s developmental status in the case of milestones or abilities that caregivers may not yet notice have developed [4]. However, children’s differential comfort with assessors may affect performance, and introduce bias if children react differently by intervention status. Children who did not receive the intervention may be more reserved with strangers and therefore a direct assessment may underestimate their true ability as compared to children who received the intervention and are more used to interacting with strangers. This bias has previously been considered to be smaller than the potential bias due to caregiver report [4], and as such direct assessments are considered to have less bias in assessment of intervention effects.

A direct assessment measure that has been used in over 20 countries globally is the Bayley Scales of Infant and Toddler Development III (Bayley-III), a tool that is administered through direct assessment for the evaluation of cognitive, motor, and language development for children between the ages of 1 and 42 months [4–6]. The Bayley-III, however, comes with a large initial cost, as well as a high cost per assessment, more extensive training compared to caregiver report, a controlled environment for administration, and a lengthy administration time [4]. Thus, the Bayley-III is difficult to use in settings where financial, human, or time resources are constrained, and can be prohibitively time and resource intensive in the case of large-scale evaluations in low-resource settings.

Ages and Stages Questionnaire (ASQ) assessments are primarily caregiver report and have been administered in over 20 LMICs [7]. These caregiver report assessments are cheaper than the Bayley-III and can be administered more quickly with less training. The version of the ASQ used most often to assess child development in low- and middle-income contexts to date is the ASQ-3 [7]. It has been translated and adapted to be used in many different contexts, including Brazil, where it was found to be appropriate for screening in daycare centers [8]. The ASQ-3 is administered primarily as a caregiver report assessment with inclusion of observation items when the caregiver is unable to answer a question. The ASQ-3 includes the administration of 6 questions for each domain that depend on the child’s age group. It was designed as a short screening tool to detect developmental delay and is used in well-baby visits.

In the last two decades the ASQ-3 has also been used to evaluate the impacts of early interventions on child development in low-income contexts. Two previous studies that examined the concurrent validity of the ASQ-3 and the Bayley-III in upper-middle-income countries, in rural China [9, 10] and urban Colombia [11], found low to medium correlations between the measures for children under 24 months of age. The ASQ-3 has been adapted for use in research studies to avoid ceiling effects that occur because of the small number of questions asked per domain, and to include direct observation of some items that caregivers might not observe. Adaptations to the ASQ-3 include the Extended Ages and Stages Questionnaire (EASQ), which includes a subset of direct assessment items and extends the number of questions asked to children in each age range by adding a few questions from both the previous and subsequent age groups. The EASQ was adapted from the ASQ-3 by researchers, and has been used to evaluate programs in multiple LMICs including Bangladesh and Kenya [12–14]. A further adaptation, the Ages and Stages Questionnaire Inventory (ASQ:I) also expands on the ASQ-3. The ASQ:I is administered as a continuous measure with starting rules that depend on the child’s age, and stopping rules that depend on the child’s performance [15]. The ASQ:I reduces the potential for the ceiling effects that are found in ASQ-3 and EASQ which limit the number of questions for each domain to 6 or 12, respectively, and explicitly enable it to be used as a progress monitoring tool in addition to a screener for developmental delay [16].

The ASQ:I has been used in the evaluation of an intervention in Madagascar and in a longitudinal cohort of children in Kenya [17, 18]. It has been adapted for use in China where it was found to have adequate psychometric properties when compared to the Beijing Gesell Development Schedule [16]. In Bangladesh, the ASQ:I was adapted by researchers to include a subset of items that are administered through direct assessment with inexpensive and common materials. Administration of the ASQ:I requires more training and a longer administration time than the ASQ-3, because of the starting and stopping rules and the subset of direct assessment items, however it can be administered more quickly with less training compared to the Bayley-III. In the ASQ:I the questions are administered in a continuous scale restricted by the child’s performance instead of blocks of questions restricted by the child’s age, and a subset of direct assessment items are included. For these reasons we hypothesized that the Bangladeshi adapted ASQ:I would have stronger concurrent validity with the Bayley-III than has previously been reported in different settings for the ASQ-3 [11, 19]. In this study we aim to assess the concurrent correlation between the Bangladeshi adapted ASQ:I against the Bayley-III in rural Bangladesh.

## Methods

### Participants

Data were collected between July and August 2018. Participants are a subset of those from the endline assessment of a cluster randomized controlled trial of an early child development intervention in Kishoreganj District, Bangladesh (the RINEW trial) [20]. Women in their second or third trimester of pregnancy or female primary caregivers of children under 15 months were randomly selected from eligible women in selected villages. Additional details on randomization have been presented in previous work [20]. At the endline assessment children with visible physical or cognitive disabilities were excluded, as was one randomly selected child for each pair of twins. At intervention endline, all children were assessed on the ASQ:I (*n* = 574 from 31 villages, 15 control and 16 intervention). For the current study, a stratified subset of 16 villages (8 control, 8 intervention) were selected for the Bayley-III assessment from those that had children of both sexes in each age group (6–12, 13–18, and 19–24 months).

### Measures

The Bayley-III assessment consists of five subscales (cognitive, gross motor, fine motor, receptive language, and expressive language) that are administered through direct assessment. During scoring, these subscales can be combined into three composite domains that are externally standardized to a US sample: cognitive, motor, and language. For this analysis we examined the raw scores on each subscale as opposed to the composite cognitive, motor and language domain scores to ensure the scores were comparable to those on the ASQ:I. This analysis is in line with previous work [9, 11]. The Bayley-III also includes two domains assessed through caregiver report (adaptive behavior and socio-emotional), which were not administered as part of this study. The Bayley-III was translated to Bengali and culturally adapted to the Bangladeshi context through the replacement of culturally inappropriate pictures without changing the order of the items or their underlying concepts. This cultural adaptation was previously validated in Bangladesh [6]. The Bayley-III served as the criterion measure in this analysis.

The adapted ASQ:I assessment for this study was first piloted by members of the study team with 50 children in the Hossainpur subdistrict of Kishoreganj, Bangladesh in 2010. During this pilot, some items were adapted to ensure they were culturally appropriate, and direct assessment using inexpensive and common materials was piloted for a subset of the questions. A version of the EASQ which used these culturally adapted questions, including the subset of direct assessment items was used in the evaluation of a water, sanitation, hygiene and nutrition intervention in rural Bangladesh [12]. The 7-day test–retest reliability of the assessment during this pilot (*n* = 28) was between 0.97–0.99 (intraclass correlation, ICC) for all domains. The direct assessment items were further piloted with 453 children in 21 villages in Keraniganj subdistrict of Dhaka district, Bangladesh [21]. The ASQ:I consists of five domains: problem solving, gross motor, fine motor, communication, and personal social. The majority of the adapted ASQ:I items were assessed through caregiver report, with 16% of items assessed through direct assessment of the child (between 8–32% depending on the domain), using low-cost stimuli (table S1; figure S1). To ensure appropriate ordering of questions in order of increasing difficulty to justify stopping rules, the translated ASQ:I was piloted on 60 non-study children between 1 and 54 months old ﻿just prior to the start of the current study. Based on the proportion of children who attained each item in each age group, items were re-ranked to earlier or later positions when relevant. Both the Bayley-III and ASQ:I have an age-based starting rule, and a stopping rule that depends on the child’s performance or reported ability.

In previous work, child development has been correlated with maternal education and stimulation in the home [14, 22]. In this study maternal education was assessed by asking the mother the number of years of education she had received. Stimulation in the home was assessed by the play activities and play materials subscales of the Family Care Indicators (FCI) [23]. The play activities subscale consists of six questions about the variety of stimulating play activities the child has participated in with an adult over the past 3 days. The questions differentiate between activities that involved the child's mother, father, or other adults in the household. We present descriptive statistics and correlations for play activities that the child participated in with their mother. The play materials subscale consists of an observation of the variety of play materials that a child has played with in the past 30 days.

### Assessments

The assessors who assessed children on the ASQ:I and Bayley-III were trained separately. ASQ:I assessors had completed a minimum of a bachelor’s degree and received 7 days of training on the tool. Bayley-III assessors had a minimum of a master’s degree and received 15 days of training. Training for both groups included interactive discussion, role play, and field testing in non-intervention sites followed by inter-observer and reliability testing, feedback, and refresher trainings. Participants were assessed in their own homes, and assessments were conducted in Bengali. During the ASQ:I assessments, inter-rater reliability between the assessors and a supervisor was conducted for 4.7% of the assessments used in this analysis (*n* = 12), and the ICC was > 0.98 for all domains. For the Bayley-III assessments inter-rater reliability data was not maintained following data collection, but feedback or correction was given immediately following the assessments that were observed. During training each Bayley-III assessor did 10 practice assessments, and the ICC was > 0.98 between assessors and the trainer for all Bayley-III domains.

### Statistical analysis

Scores for each domain on both assessments were internally standardized using local-mean standardization by age in days to the control group sample that was included in this analysis. Total ASQ:I and Bayley-III scores were created by summing raw scores across all domains before standardizing. Observations with scores greater than 4 standard deviations from the control group mean were excluded, and remaining observations were re-standardized. As a measure of internal consistency, Cronbach’s alpha was calculated with raw item scores for each domain of the ASQ:I and Bayley-III assessments. Items with no variability in the sample were excluded prior to calculation of Cronbach’s alpha.

We calculated Pearson correlations for the ASQ:I and Bayley-III assessments by domain, both across the full sample, and stratified by intervention arm (any intervention vs. control) and by child age group (4–12, 13–18 and 19–26 months). For all correlations we constructed quantile-based confidence intervals with 1000 bootstrap samples clustered at the village level. We classified correlations as low (*r* = 0.20–0.39), medium (*r* = 0.40–0.59), or high (*r* =  > 0.60) [24]. Throughout we focused the results and interpretation on correlations between subscales that assessed similar constructs across assessments (Table 1). These subtests were designed to cover similar or the same underlying constructs and so should theoretically be the most correlated across tests. We also presented correlations between each subtest of both assessments in the results tables to be consistent with prior work [9, 11]. Since we did not administer the caregiver report subtests as part of the Bayley-III assessment there is no Bayley-III subtest with a similar construct to the ASQ:I Social-emotional subtest. As such, we presented the correlation between the ASQ:I Social-emotional subtest and each of the Bayley-III subtests but did not interpret or highlight this result. We then computed the concurrent correlations between scores on each subtest for each assessment and maternal education and stimulation activities in the home, variables known to be related to child development in other work. Analyses were performed in Stata v14, and R (V.4.0.1, Vienna Austria).

**Table 1:** Characteristics of assessment tools

	**Bayley Scales of Infant and Toddler Development III (Bayley-III)**	**Ages and Stages Questionnaire-Inventory (ASQ:I)**
**Type of assessment**	Direct assessment of ability	Parent report with 16% of items direct assessment
**Cost**	$1025 kit + $4.89 per child	Contact publisher for more information
**Qualifications of assessor**	Master’s degree in psychology degree; A degree or license to practice in the healthcare or allied healthcare field; or formal training specific to assessing children. Often requires 3 or more weeks of training.	Easy to use by providers with varying levels of education and expertise. Generally requires 1–2 weeks of training
**Duration**	60–120 min	45–60 min
**Subtests assessed (similar domains shown in the same row)**	Cognitive	Problem solving
	Fine Motor	Fine Motor
	Gross Motor	Gross Motor
	Receptive Language	Communication﻿
	Expressive Language	
		Personal-Social

## Results

Of the total sample of children in the 16 villages selected for the Bayley-III assessment, 300 (*n* = 151 control; *n* = 149 intervention) received the ASQ:I assessment. A total of 244 (81%) of these children were assessed on the Bayley-III (*n* = 128 control; *n* = 116 intervention).

Children in the sample were on average 16.2 (SD 5.4) months of age, with 30% (*n* = 73), 35% (*n* = 86), and 35% (*n* = 85) in the 4–12, 13–18, and 19–26 month age groups respectively (Table 2). Female and male children each made up approximately half of the sample (45% girls, 55% boys). All primary caregivers except 3 were the child’s biological mother. Mother’s education was on average 6.4 (SD 3.2) years. Demographic characteristics did not differ across the participants sampled in the control and intervention arms. The scores for the FCI subscales and the scores on the ASQ:I and Bayley-III were higher amongst children in the intervention arm (Table 2).

**Table 2:** Characteristics of the sample

	**Total sample (*n* = 244)**	**Control (*n* = 128)**	**Intervention (*n* = 116)**
**Caregiver Characteristics**			
Age (years)^a^	25 (5.8)	25.7 (6.0)	25.0 (5.6)
Primary caregiver is biological mother^a^	99% (*n* = 240)	98% (*n* = 125)	99% (*n* = 115)
Mother’s education (years)	6.4 (3.2)	6.1 (3.4)	6.7 (3.0)
Mother completed primary education	63% (*n* = 154)	58% (*n* = 74)	69% (*n* = 80)
Father’s education (years)^b^	4.6 (4.0)	4.6 ± 3.9	4.7 (4.2)
Father completed primary education^b^	34% (*n* = 81)	34% (*n* = 41)	35% (*n* = 40)
**Child Characteristics**			
Age at Bayley-III assessment (months)	16.2 (5.4)	15.6 (5.2)	16.9 (5.4)
4–12 months	30% (*n* = 73)	36% (*n* = 42)	24% (*n* = 31)
13–18 months	35% (*n* = 86)	42% (*n* = 49)	29% (*n* = 37)
19–26 months	35% (*n* = 85)	32% (*n* = 37)	38% (*n* = 48)
Female	45% (*n* = 111)	44% (*n* = 56)	47% (*n* = 55)
FCI play activities subscale (0–6)	3.7 (1.6)	3.1 (1.5)	4.5 (1.4)
FCI play materials subscale (0-6)	2.9 (1.7)	2.2 (1.4)	3.7 (1.7)
**Household Characteristics**			
Household size (number of people)	5.5 (2.1)	5.4 (1.9)	5.7 (2.3)
Has cement floor	18% (*n* = 43)	18% (*n* = 23)	17% (*n* = 20)
Has brick walls	21% (*n* = 50)	24% (*n* = 31)	16% (*n* = 19)
Has electricity	88% (*n* = 215)	82% (*n* = 23)	95% (*n* = 110)
**Bayley-III Raw Scores**			
Cognitive^c^	46.2 (9.6)	44.8 (9.5)	47.8 (9.5)
Receptive language	17.3 (5.4)	16.3 (5.2)	18.3 (5.4)
Expressive language^a^	19.3 (6.3)	18.0 (6.0)	20.6 (6.4)
Fine motor^c^	31.4 (6.0)	30.6 (6.2)	32.3 (5.7)
Gross motor^e^	46.8 (9.7)	46.0 (9.7)	47.8 (9.6)
Total score^f^	161.4 (35.2)	156.1 (35.0)	167.0 (34.9)
**Bayley-III Composite Scores**			
Cognitive^c^	94.8 (11.7)	93.6 (13.3)	96.0 (9.7)
Language^a^	96.5 (14.4)	94.6 (16.6)	98.6 (11.3)
Motor^d^	100.0 (13.8)	99.3 (14.7)	100.7 (12.8)
**ASQ:I Raw Scores**			
Problem solving^g^	54.3 (15.9)	51.3 (15.8)	57.6 (15.4)
Communication^a^	55.7 (16.1)	52.9 (15.3)	58.8 (16.5)
Fine motor^f^	49.8 (11.5)	48.3 (11.3)	51.5 (11.5)
Gross motor^h^	58.1 (16.5)	55.9 (16.3)	60.5 (16.4)
Personal social^i^	54.0 (16.6)	50.9 (15.9)	57.5 (16.8)
Total^j^	271.8 (72.7)	260.0 (70.3)	284.4 (73.4)

Notes: Data are % (n) or mean (SD)

Maximum scores for Bayley-III domains: cognitive = 91; receptive language = 49; expressive language = 48; fine motor = 66; gross motor = 72; total = 326. Maximum scores for ASQ:I domains: problem solving = 50; communication = 63; fine motor = 63; gross motor = 65; personal social = 68; total = 309

*FCI* Family Care Indicators, *Bayley-III* Bayley Scales of Infant and Toddler Development III, *ASQ:I* Ages and Stages Questionnaire Inventory

^a^*n* = 243; ^b^*n* = 235; ^c^*n* = 241; ^d^*n* = 240; ^e^*n* = 242; ^f^*n* = 237; ^g^*n* = 239; ^h^*n* = 238; ^i^*n* = 234; ^j^*n* = 225

Assessments occurred on average two weeks apart (median 14 days, IQR 7 to 18 days). We had a total of 3 participants who had a score that was an outlier on either assessment, and 21 participants who had missing data on one or more domains, resulting in a total of between 220 and 243 participants observed on each of the ASQ:I and Bayley-III domains. The Cronbach’s alpha was between 0.77–0.81 for the ASQ:I domains, and between 0.90–0.95 for the Bayley-III domains (Table 3).

**Table 3:** Internal consistency by domain

Domain	Cronbach’s alpha
**Bayley-III**	
Cognitive	0.93
Fine Motor	0.91
Gross Motor	0.95
Receptive Language	0.90
Expressive Language	0.91
**ASQ:I**	
Problem Solving	0.78
Fine Motor	0.79
Gross Motor	0.79
Communication	0.77
Personal Social	0.81

### Concurrent validity

**Fig. 1:**
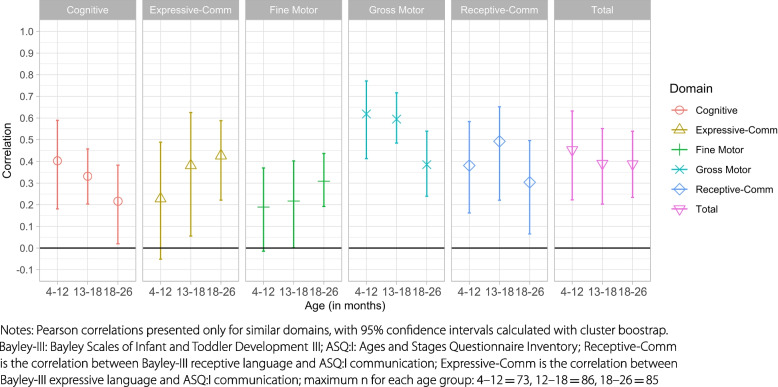
Correlation between Bayley-III and ASQ:I assessments by child age and domain

The concurrent validity for domains that assessed similar constructs ranged from 0.24 (fine motor) to 0.55 (gross motor), with a correlation between total scores of 0.42 (Table 4). Concurrent validity of the total score did not systematically differ by age, however there were suggestions of trends of increased correlation between scores by age for the fine motor and communication domains, and decreased correlation by age for the gross motor and cognitive domains (Fig. 1; table S2). Concurrent validity in the intervention group did not differ systematically from the control group across domains (Table 5). The two sets of similar domains with correlations that differed by more than 0.10 across intervention and control arms were the correlation between receptive language (Bayley-III) and communication (ASQ:I) which was higher for the intervention arm (0.44 vs. 0.32), and the correlation between gross motor scores on both measures, which was higher for the control arm (0.63 vs. 0.50).

**Table 4:** Correlation between ASQ:I and Bayley-III domains in the full sample

	**Bayley-III domain**					
**ASQ:I domain**	Cognitive	Receptive Language	Expressive Language	Fine Motor	Gross Motor	Total
	(*n* = 237)	(*n* = 243)	(*n* = 242)	(*n* = 235)	(*n* = 237)	(*n* = 220)
Problem Solving	**0.308** (**0.23**,**0.38**)	0.287 (0.14,0.41)	0.265 (0.16,0.36)	0.286 (0.14,0.40)	0.247 (0.14,0.35)	0.346 (0.23,0.44)
Communication	0.220 (0.07,0.35)	**0.398** (**0.26**,**0.51**)	**0.345** (**0.20**,**0.47**)	0.219 (0.06,0.38)	0.242 (0.10,0.36)	0.327 (0.19,0.47)
Fine Motor	0.199 (0.09,0.28)	0.209 (0.09,0.31)	0.219 (0.12,0.30)	**0.241** (**0.11**,**0.35**)	0.217 (0.03,0.35)	0.236 (0.08,0.37)
Gross Motor	0.186 (0.08,0.28)	0.206 (0.09,0.33)	0.146 (0.07,0.22)	0.165 (0.04,0.29)	**0.545** (**0.45**,**0.63**)	0.388 (0.29,0.47)
Personal Social	0.172 (0.03,0.31)	0.260 (0.12,0.38)	0.188 (0.11,0.27)	0.233 (0.12,0.33)	0.134 (0.01,0.25)	0.245 (0.13,0.36)
Total	0.291 (0.17,0.39)	0.356 (0.24,0.45)	0.292 (0.17,0.41)	0.274 (0.11,0.42)	0.342 (0.19,0.48)	**0.423** (**0.29**,**0.54**)

Notes: Estimates are Pearson correlations between the respective ASQ:I and Bayley-III domain internally standardized scores with 95% confidence intervals calculated with cluster bootstrap. Similar domains across measures are represented in bold, n’s represent the n used for each the similar domain correlation bolded in each column

*Bayley-III* Bayley Scales of Infant and Toddler Development-III, *ASQ:I* Ages and Stages Questionnaire Inventory

**Table 5:** Correlation between Bayley-III and ASQ:I assessments by intervention status and domain

	**Bayley-III domain**					
**ASQ:I domain**	Cognitive	Receptive Lang	Expressive Lang	Fine Motor	Gross Motor	Total
**Control arm**						
Problem Solving	**0.312** (**0.19**,**0.41**)	0.108 (-0.08,0.27)	0.198 (0.00,0.33)	0.268 (0.07,0.41)	0.272 (0.12,0.40)	0.339 (0.19,0.48)
Communication	0.108 (-0.14,0.33)	**0.319** (**0.07**,**0.51**)	**0.300** (**0.08**,**0.46**)	0.232 (-0.01,0.43)	0.244 (0.14,0.35)	0.270 (0.06,0.44)
Fine Motor	0.190 (0.06,0.30)	0.070 (-0.07,0.20)	0.161 (0.02,0.30)	**0.209** (**-0.01**,**0.38**)	0.246 (0.10,0.39)	0.211 (0.06,0.34)
Gross Motor	0.099 (-0.07,0.25)	0.161 (0.01,0.32)	0.115 (0.03,0.21)	0.171 (-0.04,0.35)	**0.630** (**0.55**,**0.70**)	0.377 (0.29,0.47)
Personal Social	0.061 (-0.14,0.24)	0.161 (0.01,0.29)	0.137 (0.04,0.24)	0.134 (-0.01,0.26)	0.229 (0.09,0.41)	0.192 (0.06,0.37)
Total	0.212 (0.03,0.39)	0.230 (0.04,0.39)	0.196 (0.06,0.32)	0.241 (0.02,0.44)	0.461 (0.31,0.64)	**0.375** (**0.24**,**0.56**)
**Intervention arm**						
Problem Solving	**0.281** (**0.18**,**0.37**)	0.412 (0.29,0.53)	0.286 (0.14,0.44)	0.280 (0.10,0.46)	0.253 (0.07,0.41)	0.318 (0.13,0.47)
Communication	0.290 (0.18,0.41)	**0.444** (**0.30**,**0.59**)	**0.352** (**0.16**,**0.53**)	0.182 (0.01,0.39)	0.260 (0.02,0.46)	0.340 (0.12,0.55)
Fine Motor	0.197 (0.03,0.31)	0.301 (0.16,0.43)	0.248 (0.12,0.34)	**0.260** (**0.12**,**0.39**)	0.207 (-0.09,0.39)	0.241 (-0.01,0.43)
Gross Motor	0.240 (0.11,0.38)	0.225 (0.03,0.41)	0.149 (0.02,0.26)	0.144 (-0.01,0.30)	**0.497** (**0.33**,**0.62**)	0.384 (0.24,0.51)
Personal Social	0.218 (0.07,0.40)	0.291 (0.09,0.50)	0.171 (0.06,0.30)	0.281 (0.17,0.40)	0.098 (-0.09,0.28)	0.224 (0.06,0.38)
Total	0.332 (0.22,0.46)	0.427 (0.32,0.54)	0.328 (0.10,0.50)	0.277 (0.06,0.48)	0.278 (0.04,0.47)	**0.431** (**0.22**,**0.60**)

Notes: Estimates are Pearson correlations between the respective ASQ:I and Bayley-III domain internally standardized scores with 95% confidence intervals calculated with cluster bootstrap. Similar domains across measures are represented in bold, Maximum N per group: intervention = 128; control = 116

*Bayley-III* Bayley Scales of Infant and Toddler Development-III, *ASQ:I* Ages and Stages Questionnaire Inventory

### Correlations with other variables

All domains of the Bayley-III and ASQ:I were positively correlated with maternal education and with FCI play activities and play materials subscales (Table 6). Correlations were highest, on average, between domains on both measures and FCI play materials (correlations ranged from 0.17 to 0.43) and FCI play activities (range from 0.08 to 0.37) compared with maternal education (range from 0.03 to 0.20). Most correlations between individual domains and each of these measures were statistically significant at *p* < 0.05.

**Table 6:** Correlations between Bayley-III and ASQ:I and measures of the home environment and maternal education

	**FCI: Play activities (0–6)**	**FCI: Play materials (0–6)**	**Maternal education (years)**
**Bayley-III domain**			
Cognitive	0.195 (0.03,0.28)	0.227 (0.14,0.32)	0.084 (0.00,0.17)
Fine Motor	0.106 (-0.01,0.20)	0.170 (0.03,0.30)	0.029 (-0.11,0.15)
Gross Motor	0.076 (-0.02,0.20)	0.215 (0.10,0.34)	0.115 (-0.01,0.24)
Receptive Lang	0.202 (0.06,0.32)	0.299 (0.20,0.40)	0.103 (0.01,0.21)
Expressive Lang	0.175 (0.03,0.30)	0.265 (0.14,0.40)	0.148 (0.08,0.23)
Total	0.215 (0.07,0.32)	0.345 (0.23,0.17)	0.167 (0.07,0.25)
**ASQ:I domain**			
Problem Solving	0.305 (0.15,0.43)	0.304 (0.22,0.38)	0.111 (0.00,0.21)
Fine Motor	0.247 (0.12,0.36)	0.257 (0.17,0.33)	0.187 (0.05,0.29)
Gross Motor	0.175 (0.09,0.25)	0.282 (0.17,0.38)	0.097 (-0.03,0.21)
Communication	0.298 (0.19,0.40)	0.347 (0.25,0.44)	0.185 (0.07,0.28)
Social Emotional	0.340 (0.23,0.45)	0.315 (0.20,0.42)	0.172 (0.04,0.30)
Total	0.370 (0.24,0.48)	0.428 (0.34,0.50)	0.196 (0.07,0.32)

Notes: Estimates are Pearson correlations with 95% confidence intervals calculated with cluster bootstrap

*FCI* Family Care Indicators, *Bayley-III* Bayley Scales of Infant and Toddler Development-III, *ASQ:I* Ages and Stages Questionnaire Inventory

## Discussion

The Bangladeshi adapted ASQ:I is a low-cost tool that can be feasibly administered in Bangladesh. We found moderate correlations between the adapted ASQ:I and Bayley-III assessments for the gross motor domain and total score, and low, but significant correlations between the cognitive/problem solving, language, and fine motor domains in a sample of children aged 4–27 months in rural Bangladesh. The lower correlation between the Bayley-III cognitive domain and the ASQ:I problem solving domain was expected as the ASQ:I problem solving domain only covers a subset of the cognitive domain captured in the Bayley-III. We did not find any systematic differences in correlations between ASQ:I and Bayley-III assessments by intervention group or age. We observed significant correlations between most domains of both the ASQ:I and Bayley-III and variables that have been previously shown to correlate with better child development outcomes including the variety of play activities that an adult has participated in with the child in the last 3 days, and the variety of toys that the child has played with in the last 30 days. We also found acceptable internal consistency (Cronbach’s alpha > 0.75 for all domains) for the ASQ:I in our sample.

The concurrent validity of ASQ-3 has been assessed by domain in two upper-middle-income country settings, one in urban Colombia and two in rural China (one smaller and one larger study in the Qinba mountain region) [9–11]. Our concurrent correlations with the Bayley-III were higher than those found in the studies from Colombia and China for children under 30 months for the majority of the comparisons. The study in Colombia did not recommend use of the ASQ-3 for children under 31 months of age, as they found the majority of correlations between similar domains to be below 0.25 [11]. The continuous nature of the ASQ:I, which minimizes floor and ceiling effects and allows for more variation in outcomes, and the inclusion of direct assessment of skills that are less likely to be observed in daily life may contribute to the stronger correlation with the direct assessment measure, when compared to that of the ASQ-3 in other settings. Differences in the populations included in each study may also contribute to the differences in correlations with direct assessment measures. For example, the caregivers in our sample had less education with a mean number of years of 6.4 (SD 3.2) compared to 10.3 (SD 3.4) in Colombia and 9.2 (SD 2.7) in the larger study in China (the smaller study in China did not report years of education) [9–11]. Though previous work in India found that correlations between another caregiver report measure and the Bayley-III did not to differ by caregiver education, the large differences in caregiver education by study may be indicative of other differences between the communities [25]. For example, in communities where primary caregivers often leave the child in the care of other children or relatives, they may provide less accurate reports of developmental milestones. In these cases, the correlation between caregiver report and direct assessment may be lower. The concurrent correlation between the Chinese adapted ASQ:I and the Beijing Gesell Development Schedule was assessed in 53 children between 11 and 12 months of age in an urban setting in the city of Kunshan, China [16]. Correlations were between 0.74 and 0.89 for fine and gross motor, personal-social and problem solving/adaptive domains, and 0.29 for the communication/language domain. These correlations are higher than what we found, but we note that their assessments were done by pediatricians and that some pediatricians preferred to observe the child before interviewing the caregiver. As such this comparison was not strictly between a primarily caregiver report measure and a direct assessment and the way in which it was administered. Further, the ASQI:I was compared with the Beijing Gesell Development Schedule, not with an adapted Bayley-III and therefore is not directly comparable to our work. The researchers present the ASQ:I as a promising tool for a secondary screening measure for developmental delay, but do not discuss its use to evaluate intervention effects.

We did not find systematic differences in correlations across domains between the intervention and control study arms. One disadvantage presented for conducting assessments that employ caregiver report as part of the evaluation of child development interventions is that there may be recall bias induced by the intervention [4]. This is to say that caregivers in the intervention arms may differentially report on their children’s developmental status [4]. Two reasons for this have been presented in the literature. The first is that caregivers in the control arm may underestimate children’s development status compared to those in the intervention arm because those who did not receive the intervention may be less attentive to their child’s development status, and so may not notice the achievement of milestones that are caught by caregivers who received the intervention. The second is that caregivers in the intervention arm are taught about the importance of play and child development they may be more prone to overstating their child’s developmental status as part of social desirability bias [4]. In both cases, the intervention effects would be upwardly biased. If this were true in our sample, we would expect the correlation between the Bayley-III and the ASQ:I to be lower in the intervention group. In the current study we do not observe such a pattern, as the concurrent validity of the ASQ:I does not systematically differ for children in the intervention groups compared to the control. This apparent lack of bias indicates that differential reporting of child development across intervention arms does not seem to be an issue in this work and bolsters the overall validity of using caregiver report assessments in the evaluation of early child development interventions.

Both the study in urban Colombia and the larger study in rural China found that the concurrent validity increased by age, which we were not powered to detect in our sample. We did not see consistent correlations by age across domains. We saw increased correlations between similar domains over age for fine motor and expressive communication domains, decreased correlations for the gross motor domain, and no consistent change for cognitive, receptive communication or total scores. In the study set in urban Colombia, the more pronounced differences by age may be due to the fact that the age groups were larger and included older children (6–18, 19–30 and 31–42 month age groups) [11]. The larger study in China used similar age groups to the ones in the current study (5–12, 13–18 and 19–24), and found very low correlations between similar domains for the 5–12 month age group, *r* = 0.07 to 0.34, compared to our 0.19 to 0.62. The lower correlations in the youngest age groups may have contributed to the patterns of increased correlation over age [9]. The smaller study in rural China, which looked at correlations in 5–11, 12–17, and 18–23 month age groups, also did not found a pattern of increasing correlation between the ASQ-3 and the Bayley-III domains as children got older [10]. There is additional evidence from Chile, an upper-middle-income country with high levels of education (17.7 (SD 2.6) years), that an age gradient maybe present only when the age range is extended [26]. They found that the concurrent validity of the ASQ-3 with the Bayley-III total score was 0.55, 0.56 and 0.75 for 8, 18, and 30 month old children, respectively [26]. The lack of change in correlation between total scores on both measures at 8 and 18 ﻿months is consistent with what we see in our work.

The current study contributes to the literature on the measurement of child development in LMIC contexts and has multiple strengths. It provides more information on tools that can be feasibly used in the evaluation of large-scale interventions in low-resource contexts. It also contributes to the ASQ-specific literature with information on the performance of the ASQ:I which can be compared to the concurrent validity of the ASQ-3 in previous work. Further this study allowed for comparisons of concurrent correlations between ASQ:I and Bayley-III across intervention arms, allowing us to address a common concern with caregiver report assessments in the context of an intervention.

There are a few limitations of this work. The relatively small sample size in each age group means we have limited statistical power to detect differences in correlations by child age. Previous work has not used statistical inference when comparing concurrent validity in different groups [9–11], and, as such, we too interpret the magnitude of the differences and not their statistical significance. We also did not assess children over 27 months, and so we are not able to determine the concurrent correlations between ASQ:I and Bayley-III for children across the full age range for which the tool was developed. Additionally, in this study we assume that direct assessment is more accurate in identifying underlying abilities in children. We acknowledge, however, that even with appropriate training of skilled assessors, direct assessment may be affected by the child’s current state (including hunger, shyness, and tiredness) and thus may underestimate the child’s true ability, or be biased in assessment of intervention effects [4]. Thus, though direct assessment is considered more accurate and less biased in measurement of child development outcomes following interventions, it has limitations beyond the resources required. Finally, both assessment tools were originally developed in the United States and though both were culturally adapted and piloted in rural Bangladesh, they may be biased in identifying the underlying developmental status of children in this context.

## Conclusion

The ASQ:I has several benefits. It is low-cost, can be administered by assessors who have completed a bachelor’s degree and have received a 7-day training, and has the potential to capture intervention effects following a child development intervention. Research teams should apply the tool that best correlates with culturally appropriate direct assessment, given resource constraints. We recommend that researchers compare culturally appropriate direct assessment tools to the ASQ:I and other options that are feasible in their setting. The concurrent validity of the ASQ:I in older age groups and across socio-economic gradients warrants examination in future work.

## Supplementary Information

**Additional file 1:** **Figure S1.** Materials used during ASQ:I assessment. **Table S1.** Number of direct assessment items by ASQ:I domain. **Table S2.** Correlation between Bayley-III and ASQ:I assessments by child age and domain. 

## Data Availability

The datasets used and/or analyzed during the current study available from the corresponding author on reasonable request.

## Abbreviations

ASQ: Ages and Stages Questionnaire
ASQ-3: Ages and Stages Questionnaire, 3rd edition
ASQ:I: Ages and Stages Questionnaire Inventory
Bayley-III: Bayley Scales of Infant and Toddler Development III
EASQ: Extended Ages and Stages Questionnaire
FCI: Family Care Indicators
LMICs: Low- and middle-income countries

## References

[1] Lu C, Black MM, Richter LM (2016). Risk of poor development in young children in low-income and middle-income countries: an estimation and analysis at the global, regional, and country level. Lancet Glob Health.

[2] United Nations (2015). Transforming our world: the 2030 agenda for sustainable development.

[3] Richter L, Black M, Britto P, Daelmans B, Desmond C, Devercelli A (2019). Early childhood development: an imperative for action and measurement at scale. BMJ Glob Health.

[4] Fernald LC, Prado E, Kariger P, Raikes A (2017). A toolkit for measuring early childhood development in low and middle-income countries.

[5] Bayley N (2006). Bayley scales of infant and toddler development.

[6] Pendergast LL, Schaefer BA, Murray-Kolb LE, Svensen E, Shrestha R, Rasheed MA (2018). Assessing development across cultures: invariance of the Bayley-III scales across seven international MAL-ED sites. Sch Psychol Q.

[7] Small JW, Hix-Small H, Vargas-Baron E, Marks KP (2019). Comparative use of the ages and stages questionnaires in low- and middle-income countries. Dev Med Child Neurol.

[8] Filgueiras A, Pires P, Maissonette S, Landeira-Fernandez J (2013). Psychometric properties of the Brazilian-adapted version of the ages and stages questionnaire in public child daycare centers. Early Hum Dev.

[9] Yue A, Jiang Q, Wang B, Abbey C, Medina A, Shi Y, et al. Concurrent validity of the Ages and Stages Questionnaire and the Bayley Scales of Infant Development III in China. PLoS ONE 2019;14. 10.1371/journal.pone.0221675. 10.1371/journal.pone.0221675 PMC6728026 31487302

[10] Li Y, Tang L, Bai Y, Zhao S, Shi Y (2020). Reliability and validity of the Caregiver Reported Early Development Instruments (CREDI) in impoverished regions of China. BMC Pediatr.

[11] Rubio-Codina M, Araujo MC, Attanasio O, Muñoz P, Grantham-McGregor S (2016). Concurrent validity and feasibility of short tests currently used to measure early childhood development in large scale studies. PLoS ONE.

[12] Tofail F, Fernald LC, Das KK, Rahman M, Ahmed T, Jannat KK (2018). Effect of water quality, sanitation, hand washing, and nutritional interventions on child development in rural Bangladesh (WASH Benefits Bangladesh): a cluster-randomised controlled trial. Lancet Child Adolesc Health.

[13] Stewart CP, Kariger P, Fernald L, Pickering AJ, Arnold CD, Arnold BF (2018). Effects of water quality, sanitation, handwashing, and nutritional interventions on child development in rural Kenya (WASH Benefits Kenya): a cluster-randomised controlled trial. Lancet Child Adolesc Health.

[14] Fernald LCH, Kariger P, Hidrobo M, Gertler PJ (2012). Socioeconomic gradients in child development in very young children: Evidence from India, Indonesia, Peru, and Senegal. Proc Natl Acad Sci.

[15] Clifford J, Squires J, Bricker D (2011). Ages & Stages Questionnaires: Inventory pilot version 2.3.

[16] Xie H, Clifford J, Squires J, Chen C-Y, Bian X, Yu Q (2017). Adapting and validating a developmental assessment for Chinese infants and toddlers: the ages & stages questionnaires: inventory. Infant Behav Dev.

[17] Galasso E, Weber AM, Stewart CP, Ratsifandrihamanana L, Fernald LCH (2019). Effects of nutritional supplementation and home visiting on growth and development in young children in Madagascar: a cluster-randomised controlled trial. Lancet Glob Health.

[18] Milner EM, Fiorella KJ, Mattah BJ, Bukusi E, Fernald LCH (2018). Timing, intensity, and duration of household food insecurity are associated with early childhood development in Kenya. Matern Child Nutr.

[19] Yue A, Gao J, Yang M, Swinnen L, Medina A, Rozelle S. Caregiver depression and early child development: a mixed-methods study from rural China. Front Psychol 2018;9. 10.3389/fpsyg.2018.02500. 10.3389/fpsyg.2018.02500 PMC6295552 30618931

[20] Pitchik HO, Tofail F, Rahman M, Akter F, Sultana J, Shoab AK (2021). A holistic approach to promoting early child development: a cluster randomised trial of a group-based, multicomponent intervention in rural Bangladesh. BMJ Glob Health.

[21] Lancaster GA, McCray G, Kariger P, Dua T, Titman A, Chandna J (2018). Creation of the WHO Indicators of Infant and Young Child Development (IYCD): metadata synthesis across 10 countries. BMJ Glob Health.

[22] Walker SP, Wachs TD, Grantham-McGregor S, Black MM, Nelson CA, Huffman SL (2011). Inequality in early childhood: risk and protective factors for early child development. Lancet Lond Engl.

[23] Hamadani JD, Tofail F, Hilaly A, Huda SN, Engle P, Grantham-McGregor SM (2010). Use of family care indicators and their relationship with child development in Bangladesh. J Health Popul Nutr.

[24] Evans JD. Straightforward statistics for the behavioral sciences. Thomson Brooks/Cole Publishing Co; 1996.

[25] Alderman H, Friedman J, Ganga P, Kak M, Rubio-Codina M (2020). Assessing the performance of the Caregiver Reported Early Development Instruments (CREDI) in rural India. Ann N Y Acad Sci.

[26] Schonhaut L, Armijo I, Schönstedt M, Alvarez J, Cordero M (2013). Validity of the ages and stages questionnaires in term and preterm infants. Pediatrics.

