# Supplementary material for: Concurrent validity of the Ages and Stages Questionnaire Inventory and the Bayley Scales of Infant and Toddler Development in rural Bangladesh
Source: BMC Pediatr. 2023 Mar 1;23:93. doi: 10.1186/s12887-022-03800-6 (PMC9976496; doi:10.1186/s12887-022-03800-6)
Supplement: Supplementary file 1 — Additional file 1: Figure S1. Materials used during ASQ:I assessment. Table S1. Number of direct assessment items by ASQ:I domain. Table S2. Correlation between Bayley-III and ASQ:I assessments by child age and domain. [file 12887_2022_3800_MOESM1_ESM.docx]

**Concurrent Validity of the Ages and Stages Questionnaire Inventory and the Bayley Scales of Infant and Toddler Development in Rural Bangladesh**

*Supplementary material*

**Figure S1. Materials used during ASQ:I assessment**


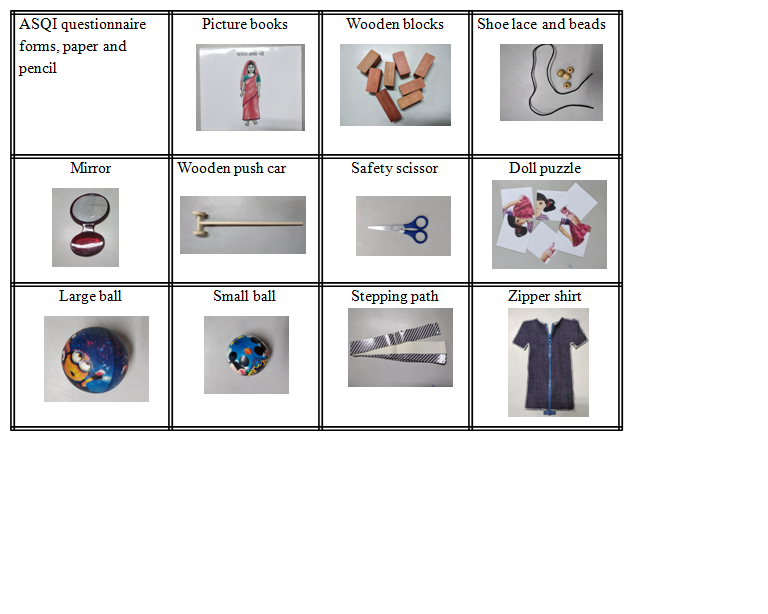


ASQ:I: Ages and Stages Questionnaire Inventory

**Table S1. Number of direct assessment items by ASQ:I domain**

| **Domain** | **Direct assessment items** | **Total items** | **%** |
| --- | --- | --- | --- |
| Communication | 6 | 64 | 9.4% |
| Gross Motor | 11 | 65 | 16.9% |
| Fine Motor | 20 | 63 | 31.7% |
| Problem Solving | 9 | 68 | 13.2% |
| Personal Social | 4 | 50 | 8% |
| **Total** | **50** | **310** | **16%** |

ASQ:I: Ages and Stages Questionnaire Inventory

**Table S2. Correlation between Bayley-III and ASQ:I assessments by child age and domain**

|  | **BAYLEY-III domain** | | | | | |
| --- | --- | --- | --- | --- | --- | --- |
|  | Cognitive | Receptive Language | Expressive Language | Fine Motor | Gross Motor | Total |
| **ASQ:I domain** |  |  |  |  |  |  |
| **4-12 months** |  |  |  |  |  |  |
| Problem Solving | **0.403** (**0.18**,**0.59**) | 0.335 (0.14,0.50) | 0.297 (0.02,0.54) | 0.198 (-0.06,0.41) | 0.299 (0.04,0.54) | 0.417 (0.16,0.62) |
| Communication | 0.280 (0.11,0.45) | **0.381** (**0.16**,**0.58**) | **0.228** (**-0.05**,**0.49**) | 0.192 (-0.02,0.41) | 0.200 (-0.03,0.44) | 0.324 (0.10,0.53) |
| Fine Motor | 0.178 (-0.05,0.39) | 0.254 (0.00,0.42) | 0.257 (0.12,0.39) | **0.189** (**-0.01**,**0.37**) | 0.255 (0.00,0.52) | 0.303 (0.09,0.49) |
| Gross Motor | 0.168 (-0.07,0.38) | 0.150 (-0.02,0.33) | 0.197 (0.04,0.36) | 0.078 (-0.08,0.24) | **0.619** (**0.41**,**0.77**) | 0.420 (0.21,0.61) |
| Personal Social | 0.064 (-0.18,0.28) | 0.370 (0.09,0.59) | 0.215 (0.02,0.42) | 0.243 (-0.01,0.46) | 0.196 (-0.06,0.45) | 0.291 (0.00,0.54) |
| Total | 0.270 (0.17,0.39) | 0.422 (0.19,0.59) | 0.293 (0.08,0.50) | 0.212 (0.01,0.41) | 0.401 (0.14,0.66) | **0.453** (**0.22**,**0.63**) |
| **13-18 months** |  |  |  |  |  |  |
| Problem Solving | **0.332** (**0.20**,**0.46**) | 0.326 (-0.01,0.56) | 0.260 (-0.02,0.49) | 0.236 (-0.02,0.44) | 0.078 (-0.15,0.32) | 0.335 (0.13,0.49) |
| Communication | 0.084 (-0.07,0.27) | **0.492** (**0.22**,**0.65**) | **0.381** (**0.06**,**0.63**) | 0.193 (-0.15,0.45) | 0.159 (-0.04,0.36) | 0.311 (0.11,0.49) |
| Fine Motor | 0.194 (0.04,0.36) | 0.196 (-0.09,0.39) | 0.243 (0.02,0.46) | **0.217** (**0.00**,**0.40**) | 0.110 (-0.17,0.33) | 0.239 (0.04,0.40) |
| Gross Motor | 0.076 (-0.12,0.26) | 0.247 (-0.01,0.49) | 0.101 (-0.04,0.24) | 0.118 (-0.17,0.36) | **0.595** (**0.48**,**0.72**) | 0.384 (0.26,0.50) |
| Personal Social | 0.140 (-0.04,0.38) | 0.245 (-0.01,0.41) | 0.206 (-0.01,0.38) | 0.252 (0.01,0.42) | 0.036 (-0.21,0.32) | 0.212 (0.02,0.37) |
| Total | 0.250 (0.08,0.43) | 0.385 (0.06,0.59) | 0.317 (0.04,0.53) | 0.274 (0.01,0.41) | 0.228 (0.01,0.48) | **0.391** (**0.20**,**0.55**) |
| **19-26 months** |  |  |  |  |  |  |
| Problem Solving | **0.216** (**0.02**,**0.38**) | 0.201 (-0.01,0.37) | 0.259 (0.17,0.33) | 0.419 (0.28,0.54) | 0.343 (0.21,0.45) | 0.286 (0.15,0.46) |
| Communication | 0.254 (0.01,0.42) | **0.304** (**0.07**,**0.50**) | **0.426** (**0.22**,**0.59**) | 0.268 (0.04,0.48) | 0.321 (0.11,0.48) | 0.305 (0.07,0.54) |
| Fine Motor | 0.214 (0.09,0.31) | 0.166 (-0.03,0.29) | 0.173 (0.03,0.27) | **0.309** (**0.19**,**0.44**) | 0.255 (0.06,0.38) | 0.155 (-0.05,0.33) |
| Gross Motor | 0.255 (0.03,0.43) | 0.179 (0.01,0.34) | 0.107 (-0.09,0.25) | 0.305 (0.15,0.45) | **0.385** (**0.24**,**0.54**) | 0.324 (0.14,0.48) |
| Personal Social | 0.257 (0.02,0.47) | 0.139 (-0.08,0.33) | 0.146 (-0.01,0.29) | 0.182 (-0.01,0.39) | 0.157 (-0.08,0.35) | 0.207 (0.01,0.41) |
| Total | 0.315 (0.12,0.47) | 0.186 (0.02,0.33) | 0.269 (0.08,0.41) | 0.336 (0.22,0.49) | 0.365 (0.22,0.53) | **0.389** (**0.23**,**0.54**) |

Notes: Concordant domains across measures are represented in bold; Maximum n per age group: 4-12=73; 13-18=86; 19-26=85

Bayley-III: Bayley Scales of Infant and Toddler Development-III; ASQ:I: Ages and Stages Questionnaire Inventory
